# Not Lower-Limb Joint Strength and Stiffness but Vertical Stiffness and Isometric Force-Time Characteristics Correlate With Running Economy in Recreational Male Runners

**DOI:** 10.3389/fphys.2022.940761

**Published:** 2022-06-28

**Authors:** Qin Zhang, George P. Nassis, Shiqin Chen, Yue Shi, Fei Li

**Affiliations:** ^1^ School of Physical Education and Sport Training, Shanghai University of Sport, Shanghai, China; ^2^ Physical Education Department, College of Education, United Arab Emirates University, Al Ain, United Arab Emirates; ^3^ Department of Sports Science and Clinical Biomechanics, SDU Sport and Health Sciences Cluster (SHSC), University of Southern Denmark, Odense, Denmark; ^4^ School of Kinesiology, Shanghai University of Sport, Shanghai, China

**Keywords:** neuromuscular characteristics, isometric mid-thigh pull, running performance, eccentric strength, stiffness, recreational runner

## Abstract

Neuromuscular characteristics, such as lower-limb joint strength, the ability to reuse elastic energy, and to generate force are essential factors influencing running performance. However, their relationship with running economy (RE) remains unclear. The aim of this study was to evaluate the correlations between isokinetic lower-limb joint peak torque (PT), lower-limb stiffness, isometric force-time characteristics and RE among recreational-trained male runners. Thirty male collegiate runners (aged 20–22 years, VO_2max_: 54.02 ± 4.67 ml·kg^−1^·min^−1^) participated in test sessions on four separate days. In the first session, the body composition and RE at 10 km·h^−1^ were determined. In the second session, leg and vertical stiffness (K_leg_ and K_vert_), knee and ankle stiffness (K_knee_ and K_ankle_) were evaluated. In the third session, isokinetic knee and ankle joint PT at velocity of 60°s^−1^ were tested. The force-time characteristics of isometric mid-thigh pull (IMTP) were evaluated in the final session. The Pearson’s product-moment correlations analysis shows that there were no significant relationships between knee and ankle joint concentric and eccentric PT, K_knee_ and K_ankle_, K_leg_, and RE at 10 km·h^−1^. However, K_vert_ (r = −0.449, *p* < 0.05) and time-specific rate of force development (RFD) for IMTP from 0 to 50 to 0–300 ms (r = −0.434 to −0.534, *p* < 0.05) were significantly associated with RE. Therefore, superior RE in recreational runners may not be related to knee and ankle joint strength and stiffness. It seems to be associated with vertical stiffness and the capacity to rapidly produce force within 50–300 ms throughout the lower limb.

## Introduction

The running economy (RE) is defined as the steady-state oxygen or energy expenditure at a given running speed and is an essential physiological contributor to distance running success (Blagrove et al., 2018). RE reflects the energy demand during constant submaximal running and is cited as a stronger indicator of endurance performance than maximum oxygen uptake (VO_2max_) among a homogenous group of runners with similar aerobic capacity (Conley and Krahenbuhl, 1980; Morgan et al., 1989). Thus, a superior RE means that runners use less energy to sustain a given steady-state speed. Given the critical contribution of RE to running performance, exploring the principal factors of RE appears essential. Neuromuscular strength is an important factor in the determination of RE, and many studies have indicated that improvement in RE following strength training interventions is attributed to neuromuscular adaptation, such as a consequence of changes in lower-limb muscle strength, stiffness and power (Barnes and Kilding, 2015; Blagrove et al., 2018; Trowell et al., 2020).

Lower limb muscles, especially those at the knee and ankle play an important role in running (Hamner et al., 2010; Hamner and Delp, 2013). For example, during the stance phase, the knee extensor muscles are the main contributors to decelerate and support the center of mass (COM) at the braking phase (Hamner et al., 2010; Hamner and Delp, 2013) and allow the force production in the knee flexor muscles during the propulsive phase (Liu et al., 2008; Monte et al., 2020). The ankle plantar flexor muscles are also activated during running, generating force more than 12 times the body weight (Komi, 1990), and are the most dominant contributors to force during the propulsion phase (Hamner et al., 2010; Monte et al., 2020). Meanwhile, ankle plantar flexor muscles perform quasi-isometric contractions while generating muscular contraction forces during the braking phase and approach their optimal contraction length, thus facilitating greater displacement of the Achilles tendon for stretch and recoil, which facilitates the storage and recovery of elastic potential energy (Farris and Sawicki, 2012; Lai et al., 2014; Monte et al., 2020). Similarly, the knee extensor muscles also perform quasi-isometric behavior and near-optimal length during the propulsion phase (Bohm et al., 2018; Monte et al., 2020), which allows for more economical production of muscle force (Fletcher and MacIntosh, 2017; Monte et al., 2020). Therefore, neuromuscular characteristics, such as knee and ankle joint muscle strength, may play an important role in improving RE.

However, the relationship between lower-limb joint strength and RE remains unclear. For instance, Sundby and Gorelick. (2014) found no significant relationship between knee flexor, extensor muscles peak torque (PT) and RE in female runners, whereas Westblad et al. (1996) found a modest correlation between eccentric knee extensor strength and RE in male distance runners. In the aspect of ankle joint strength, Bohm et al. (2021) reported that an increase in plantar flexor muscles (i.e., soleus) strength reduces the energy cost of running. To our knowledge, none of the studies have directly investigated the association between ankle joint strength and RE. Therefore, the scientific literature requires a comprehensive investigation of the correlation between lower-limb joint strength and RE.

In athletic performance tasks, lower-limb stiffness is commonly reflected by vertical stiffness (K_vert_), leg stiffness (K_leg_) and joint stiffness (Maloney and Fletcher, 2021). These measures are identified by different methods, including the spring-mass (K_vert_ and K_leg_) and torsional spring model (joint stiffness). K_vert_ and K_leg_ were considered to be contributors to the improvement of RE by reducing energy cost in vertical movements (Heise and Martin, 2001) and increasing the elastic energy storage capacity of the leg muscle-tendon units (Dalleau et al., 1998). However, the results of the existing studies seem contradictory. For example, Man et al. (2016) indicated that both K_vert_ and K_leg_ were significantly correlated with RE, whereas Heise and Martin. (1998) found that K_vert_ was moderately correlated with RE, while K_leg_ was not. Furthermore, the lower-limb joints constitute a multi-spring system with different elasticities and viscosities (Kuitunen et al., 2002). The torsional spring model provides a different perspective on spring-mass model, while knee or ankle stiffness (K_knee_ and K_ankle_) has the greatest effect on leg-spring stiffness during running (Struzik et al., 2021). However, few studies have examined the associations of K_knee_ and K_ankle_ with RE. To date, only Tam et al. (2018) reported that greater K_knee_ and lesser K_ankle_ were significantly associated with better RE in well-trained runners. Therefore, the comprehensive investigation of the relationship between K_vert_, K_leg_ and joint stiffness, and RE were essential to facilitate the running performance.

The runner’s capabilities to develop force and power within the transition period during landing could improve RE (Lum et al., 2020). The isometric mid-thigh pull (IMTP) is an accurate and reliable test that measures force-time characteristics, such as peak force (PF) generation and rate of force development (RFD) across various specific times (Wang et al., 2016; Brady et al., 2019). Running involves multi-joint movements and muscle activation (Novacheck, 1998), and IMTP enables the hip, knee and ankle joints to be held at a relatively specific angle during running. Therefore, it is essential to determine the association between the whole lower limb force-generating ability and RE. Currently, only Lum et al. (2020) have reported a relationship between IMTP characteristics and RE. They found that RFD of 0–100 to 0–200 milliseconds (ms) were significantly correlated with RE at 12 km·h^−1^ in recreational runners. Noticeably, the time that muscles are activated during the contact phase is 200–350 ms at submaximal speeds (10–16 km·h^−1^) (Gómez-Molina et al., 2017; García-Pinillos et al., 2019a). Therefore, it is important to investigate the RFD over 200 ms in order to have a better understanding of the relationship between force-time characteristics and RE.

To summary, regarding recreational runners, the benefits of neuromuscular factors on the RE have not been sufficiently studied (Sundby and Gorelick, 2014; Silva et al., 2018; Lum et al., 2020). As the increasing number of recreational runners worldwide, identifying these key neuromuscular profiles may benefit runners as this knowledge could be applied to improve RE and running performance through a more optimal strength training program design. The aim of this study was to evaluate the relationship between neuromuscular characteristics and RE in recreational male runners. We hypothesised that knee and ankle joint strength, lower-limb stiffness (K_vert_, K_leg_, K_knee_, and K_ankle_), and IMTP force-time characteristics (PF and RFD at 0–50 to 0–350 ms) would be significantly associated with RE.

## Materials and Methods

### Subjects

Thirty recreational-trained male runners (aged 20–22 years, VO_2max_: 54.02 ± 4.67 ml·kg^−1^·min^−1^) from the collegiate running club, who had previously competed in 5- to 21-km races at the collegiate level and had a minimum of 2 years of distance running experience, volunteered for this study. The sample size also satisfied the power requirement of correlation bivariate normal model with G*power 3.1 software (Universitat Dusseldorf, Dusseldorf, Germany), using the setting two-sided test, correlation ρ H1 = 0.5, α err prob = 0.05, Power (1-β err prob) = 0.80, Correlation ρ H0 = 0 (Cohen, 1988; Faul et al., 2009). The subjects’ basic information and physical characteristics are presented in Tables 1. Before the study, all subjects completed the Physical Activity Readiness Questionnaire (PAR-Q), and they respond “no” to all questions. Moreover, they received at least 1 year of training in long-distance running (5–21 km) and ran 20–30 km per week for 3 months prior to the study. Each subject was fully informed about the potential risks and procedures of the experiment and signed an informed consent document. This study was approved by Ethics Committee of Shanghai University of Sport, China (ID number: 2,017,047).

**TABLE 1:** Physical, physiological, and neuromuscular characteristics of the participants (*n* = 30).

Variable	Mean ± SD	Variable	Mean ± SD
***Physical and physiological characteristics***		K_leg_ at 10 km·h^−1^ (kN·m^−1^)	13.04 ± 1.49
Age (years)	21 ± 1	K_knee_ at 10 km·h^−1^ (N·m·deg^−1^)	5.64 ± 5.17
Height (cm)	180 ± 6	K_ankle_ at 10 km·h^−1^ (N·m·deg^−1^)	15.82 ± 8.23
Weight (kg)	72.11 ± 9.27	***Isokinetic strength characteristics***	
BMI (kg·m^−2^)	22.30 ± 1.88	K_flex-con_ at 60°s^−1^ (N·m·kg^−1^)	1.79 ± 0.24
FFM (kg)	60.53 ± 6.44	K_ex-con_ at 60°s^−1^ (N·m·kg^−1^)	3.20 ± 0.56
FM (kg)	11.58 ± 3.86	K_flex-ecc_ at 60°s^−1^ (N·m·kg^−1^)	2.03 ± 0.26
VO_2max_ (ml·kg^−1^·min^−1^)	54.02 ± 4.67	K_ex-ecc_ at 60°s^−1^ (N·m·kg^−1^)	3.65 ± 0.75
RER at 10 km·h^−1^	0.92 ± 0.08	A_dors-con_ at 60°s^−1^ (N·m·kg^−1^)	0.50 ± 0.08
HR at 10 km·h-1 (beats·min^−1^)	156.0 ± 13.5	A_plan-con_ at 60°s^−1^ (N·m·kg^−1^)	1.73 ± 0.24
RE at 10 km·h^−1^ (ml·kg^−1^·min^−1^)	40.60 ± 3.03	A_dors-ecc_ at 60°s^−1^ (N·m·kg^−1^)	0.81 ± 0.09
***Biomechanical characteristics***		A_plan-ecc_ at 60°s^−1^ (N·m·kg^−1^)	3.02 ± 0.53
Lower limb length (m)	0.93 ± 0.04	***Isometric force-time characteristics***	
Δy at 10 km·h^−1^ (cm)	7.44 ± 0.88	PF (N·kg^−1^)	24.44 ± 4.08
ΔL at 10 km·h^−1^ (cm)	14.29 ± 1.42	RFD_0–50_ (N·s^−1^)	6,823.35 ± 3,232.48
Tc at 10 km·h^−1^ (s)	0.25 ± 0.02	RFD_0–100_ (N·s^−1^)	6,902.45 ± 2,132.29
vGRF at 10 km·h^−1^ (N)	1857.42 ± 237.08	RFD_0–150_ (N·s^−1^)	6,829.14 ± 1,416.22
ΔM_knee_ at 10 km·h^−1^ (N·m)	139.99 ± 125.84	RFD_0–200_ (N·s^−1^)	6,406.86 ± 1,278.98
Δθ_knee_ at 10 km·h^−1^ (deg)	24.53 ± 4.27	RFD_0–250_ (N·s^−1^)	5,266.01 ± 851.16
ΔM_ankle_ at 10 km·h^−1^ (N·m)	179.80 ± 55.82	RFD_0–300_ (N·s^−1^)	4,469.71 ± 643.21
Δθ_ankle_ at 10 km·h^−1^ (deg)	13.15 ± 5.46	RFD_0–350_ (N·s^−1^)	3,982.25 ± 610.72
K_vert_ at 10 km·h^−1^ (kN·m^−1^)	25.07 ± 2.54		

BMI, body mass index; FFM, fat-free mass; FM, fat mass; VO_2max_, maximum oxygen uptake; RER, respiratory exchange ratio; HR, heart rate; RE, running economy; Δy, center of mass vertical displacement during stance phase; ΔL, change in leg length during stance phase; Tc, ground contact time; vGRF, vertical ground reaction force; ΔM_knee_, amount of change in knee joint moment from touchdown to maximum flexion; Δθ_knee_, knee joint angular displacement from touchdown to maximum flexion; ΔM_ankle_, amount of change in ankle joint moment from touchdown to maximum flexion; Δθ_ankle_, ankle joint angular displacement from touchdown to maximum flexion; K_vert_, vertical stiffness; K_leg_, leg stiffness; K_knee_, knee joint stiffness; K_ankle_, ankle joint stiffness; K_flex-con_ PT, knee flexor muscles relative peak torque in concentric action; K_ex-con_ PT, knee extensor muscles relative peak torque in concentric action; K_flex-ecc_ PT, knee flexor muscles relative peak torque in eccentric action; K_ex-ecc_ PT, knee extensor muscles relative peak torque in eccentric action; A_dors-con_ PT, dorsiflexor muscles relative peak torque in concentric action; A_plan-con_ PT, plantar flexor muscles relative peak torque in concentric action; A_dors-ecc_ PT, dorsiflexor muscles relative peak torque in eccentric action; A_plan-ecc_ PT, plantar flexor muscles relative peak torque in eccentric action; PF, relative peak force; RFD, rate of force development; RFD_0–50_, RFD, 0–50 ms; RFD_0–100_, RFD, 0–100 ms; RFD_0–150_, RFD, 0–150 ms; RFD_0–200_, RFD, 0–200 ms; RFD_0–250_, RFD, 0–250 ms; RFD_0–300_, RFD, 0–300 ms; RFD_0–350_, RFD, 0–350 ms.

### The Experimental Approach to the Problem

**FIGURE 1:**
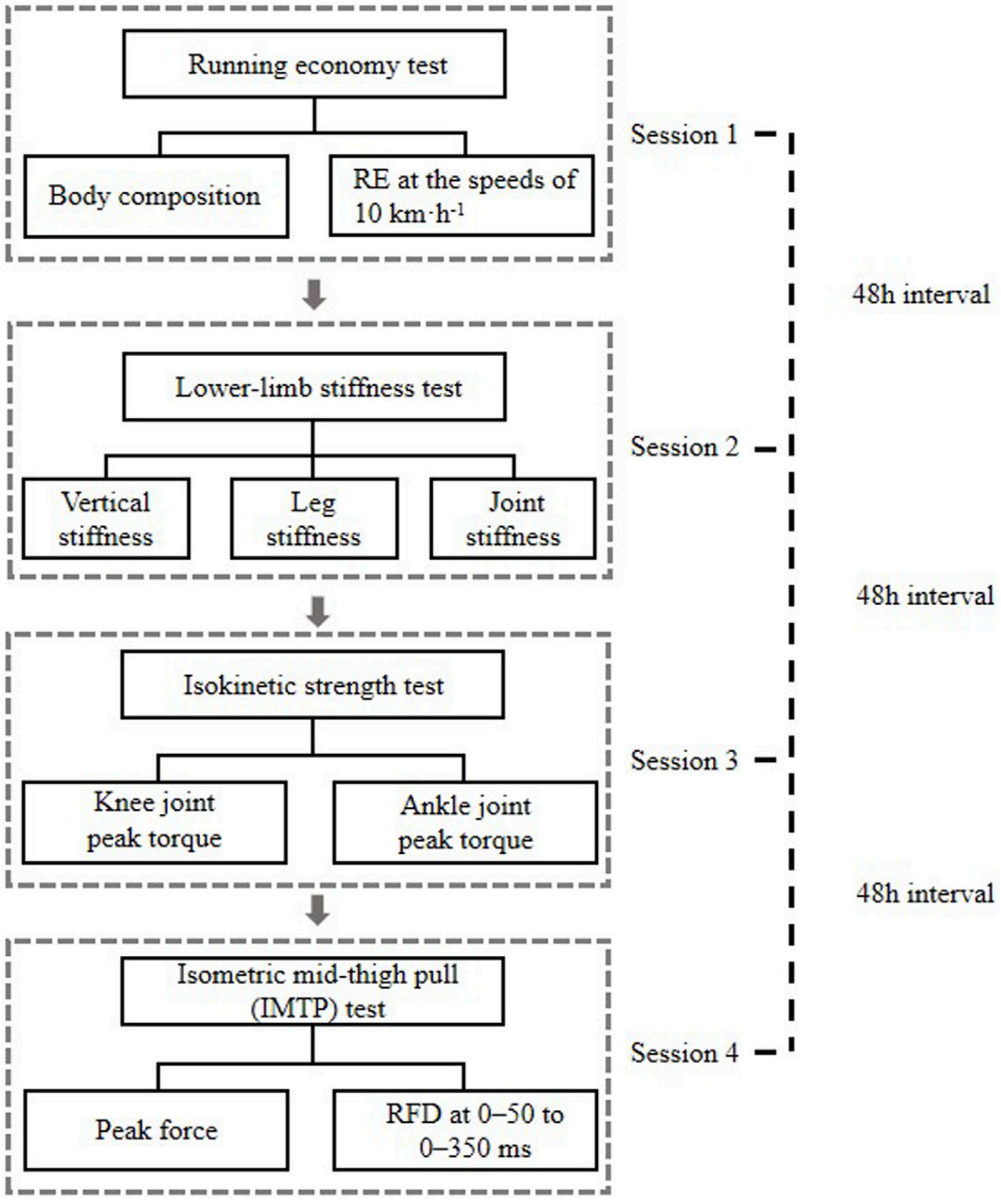
Tests flow diagram. RE, running economy; RFD, rate of force development; IMTP, isometric mid-thigh pull.

Each participant performed four separate test sessions in the laboratory with a rest interval of at least 48 h. The tests flowchart is shown in Figure 1. Before each session, they were instructed to achieve a non-fatigue state (more than 8 h of adequate sleep, no vigorous exercise for 24 h, and no muscle soreness and fatigue). The researcher examined the participants’ commitment to the instructions with a questionnaire when they reported to the laboratory for testing. In the first session, body composition and RE at the speeds of 10 km·h^−1^ were determined. In the second session, the lower-limb stiffness (K_vert_, K_leg_, K_knee_, and K_ankle_) at a speed of 10 km·h^−1^ was calculated. In the third session, the isokinetic knee and ankle joint concentric and eccentric PT at 60°s^−1^ velocity were tested, while the force-time characteristics of the IMTP (PF and RFD at 0–50 to 0–350 ms) were evaluated in the final session. Pearson’s correlation coefficients were used to investigate the possible relationships.

### Procedures

#### Body Composition Test

On the measurement day, participants were required to avoid drinking and food intake during the 3 h before testing. The weight, fat mass and fat-free mass were determined by using bioimpedance analyser (X-scan Plus II; Jawon, Korea). Standing height was recorded with a wall-mounted measuring device (Butterfly, Shanghai, China) and the body mass index (BMI) was calculated.

#### Running Economy Test

Running economy was determined using a treadmill protocol, which showed high intraclass correlation coefficient values (ICC), ranging from 0.92 to 0.94 in our laboratory (Li et al., 2021). The participants warmed up on the treadmill at a running speed of 8 km·h^−1^ for 4 min. After a 5-min rest, they ran at 10 km·h^−1^ for 4 min to determine RE, which was defined as the mean oxygen uptake (ml·kg^−1^·min^−1^) during the last minute, and the steady-state condition is verified by the respiratory exchange ratio (RER) is <1 of present subjects (Barnes and Kilding, 2015). The submaximal speed is set at 10 km·h^−1^ because this pace is similar to that used in previous study and reflects the runners’ ability to run at submaximal speeds (Piacentini et al., 2013). Oxygen uptake and heart rate (HR) were continuously monitored using a portable metabolic analyser (K5, Cosmed, Italy) and a HR monitor belt (Garmin, Olathe, United States).

#### Lower-Limb Stiffness Test

**FIGURE 2:**
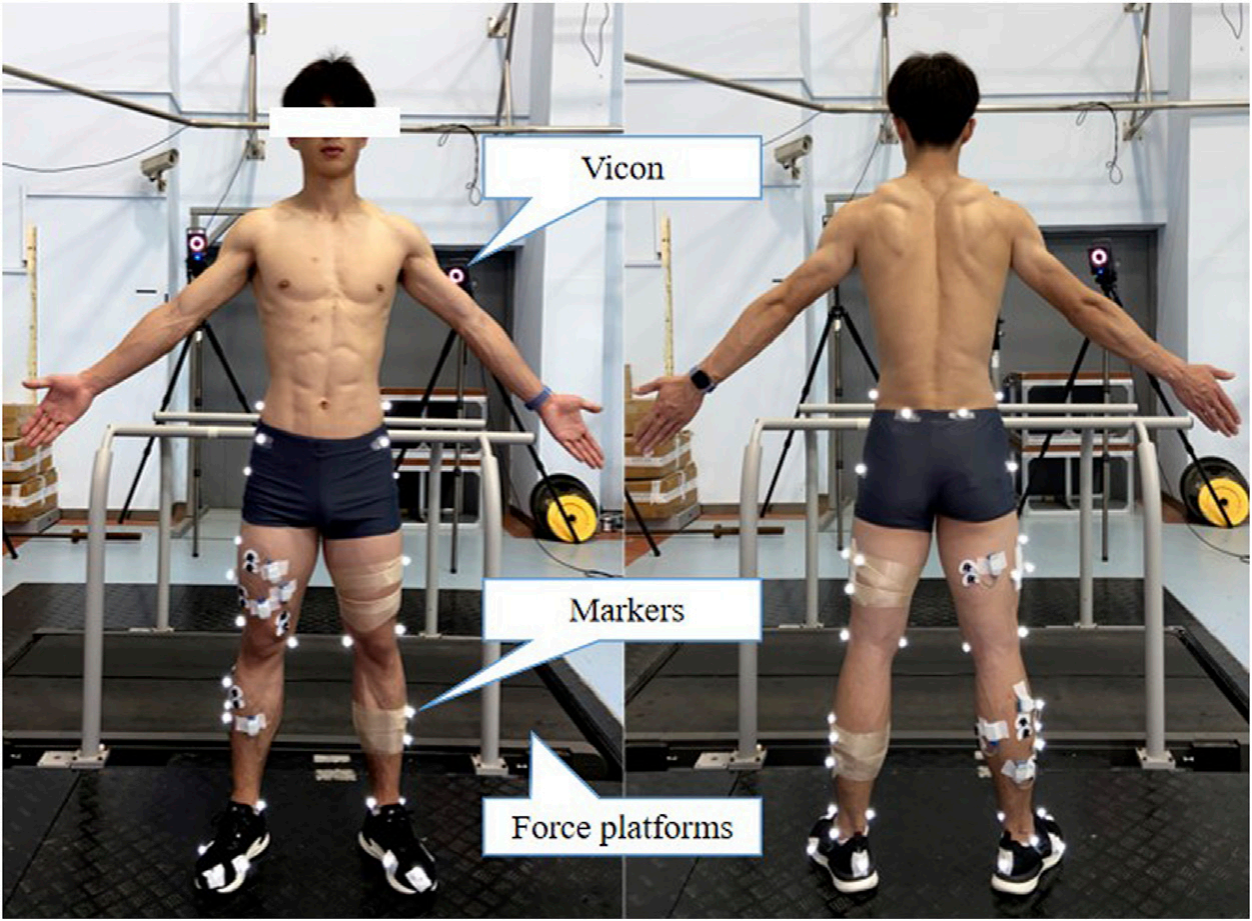
Placement of reflective markers and experimental site. The reflective markers are placed at the anterior superior iliac spine, superior margin of the iliac crest, posterior superior iliac spine, medial and lateral femoral condyles, medial and lateral malleolus, first and fifth metatarsal heads, toe and heel to identify the hip, knee and ankle joints. The markers on the T stand are used to track the trajectory of the thigh and shank.

Each participant wore identical running shoes and tight pants provided by the research team before the test, avoiding the effect of this variable on the lower-limb stiffness (Kulmala et al., 2018). Kinematic data were captured using an 8-camera Vicon T40 motion analysis system (Oxford Metrics, Oxford, United Kingdom). Ground reaction force (GRF) data was collected using two 90 cm × 60 cm × 10 cm force platforms (9287 B, Kistler Corporation) with a sampling frequency of 1,000 Hz, synchronised with the motion analysis system. The force platforms are located underneath the treadmill belt. As shown in Figure 2, 36 retroreflective markers were placed in the pelvis and lower limbs to define the foot, shank, thigh and pelvic segments. A standing calibration was recorded to identify the length of each segment and leg, the local coordinate system, and the position of the joint centre for each participant. Participants were then instructed to run on a treadmill at 8 km·h^−1^ for 4 min to warm up. After warming up, the subjects performed running for 4 min at a speed consistent with the RE test (10 km·h^−1^), during which biomechanical data collected were more valid due to being in a steady state (RER <1) (Tam et al., 2018). According to previous studies, for healthy adults, recording more than six strides is sufficient to obtain representative data, which is defined as a 95% confidence interval and an error within 5% (Besser et al., 1999). Our study took data from 10 consecutive steps starting at the third minute of running at 10 km·h^−1^ and averages were calculated for further analysis. The start and end of the support period is determined using a vertical force signal of 50 N (Kyröläinen et al., 2001).

Biomechanical data were processed by Visual 3-dimensional (3D) gait analysis software (v5, C-Motion, Inc., Germantown, MD, United States). K_vert_ and K_leg_ measurements using the sine-wave method on a treadmill showed high ICC (0.99 and 0.86) (Pappas et al., 2014). They were calculated according to the recommendations of Morin et al. (2005). K_vert_ is defined as the ratio of the maximum vertical GRF to the COM vertical displacement (Farley and González, 1996), as in Eq. 1. The K_leg_ is defined as the ratio of the maximum vertical GRF to the change in leg length (Farley and González, 1996), as in Eq. 2. In addition, Lorimer et al. (2018) confirmed that K_knee_ and K_ankle_ obtained on a treadmill exhibiting an ICC between 0.75 and 0.90. Based on previous researches, joint stiffness (knee and ankle) is interpreted as the ratio of the change in moment to the angular displacement from touchdown to when the joint is flexed to its maximum angle (Hamill et al., 2014; Tam et al., 2018), as in Eq. 3.

$$K_{vert}=\frac{F_{max}}{\Delta y}$$ (1)

where F_max_ is the maximum vertical GRF, and ∆y denotes the COM vertical displacement during stance phase.

$$\begin{array}{l} K_{leg}=\frac{F_{max}}{\Delta L},\\ \Delta L=L-\sqrt{L^{2}-{\left(\frac{vt_{c}}{2}\right)}^{2}}+\Delta y \end{array}$$ (2)

where F_max_ is the maximum vertical GRF, and ΔL denotes the change in leg length during ground contact. L is leg length in static stance (the distance from the greater trochanter of the femur to the ankle), ∆y is the COM vertical displacement during stance phase, v is the running speed (m·s^−1^), and t_c_ denotes the ground contact time (s).

$$joint\;stiffness=\frac{\Delta M}{\Delta \theta }$$ (3)

where ΔM is the amount of change in joint moment, and Δθ denotes the articular angular displacement from touchdown to maximum flexion. K_knee_ is calculated as the ratio of the change in knee moment to the angular displacement from touchdown to maximum knee flexion, and K_ankle_ is calculated as the ratio of the change in ankle moment to the angular displacement from touchdown to maximum ankle dorsiflexion in midstance.

#### Isokinetic Strength Test

**FIGURE 3:**
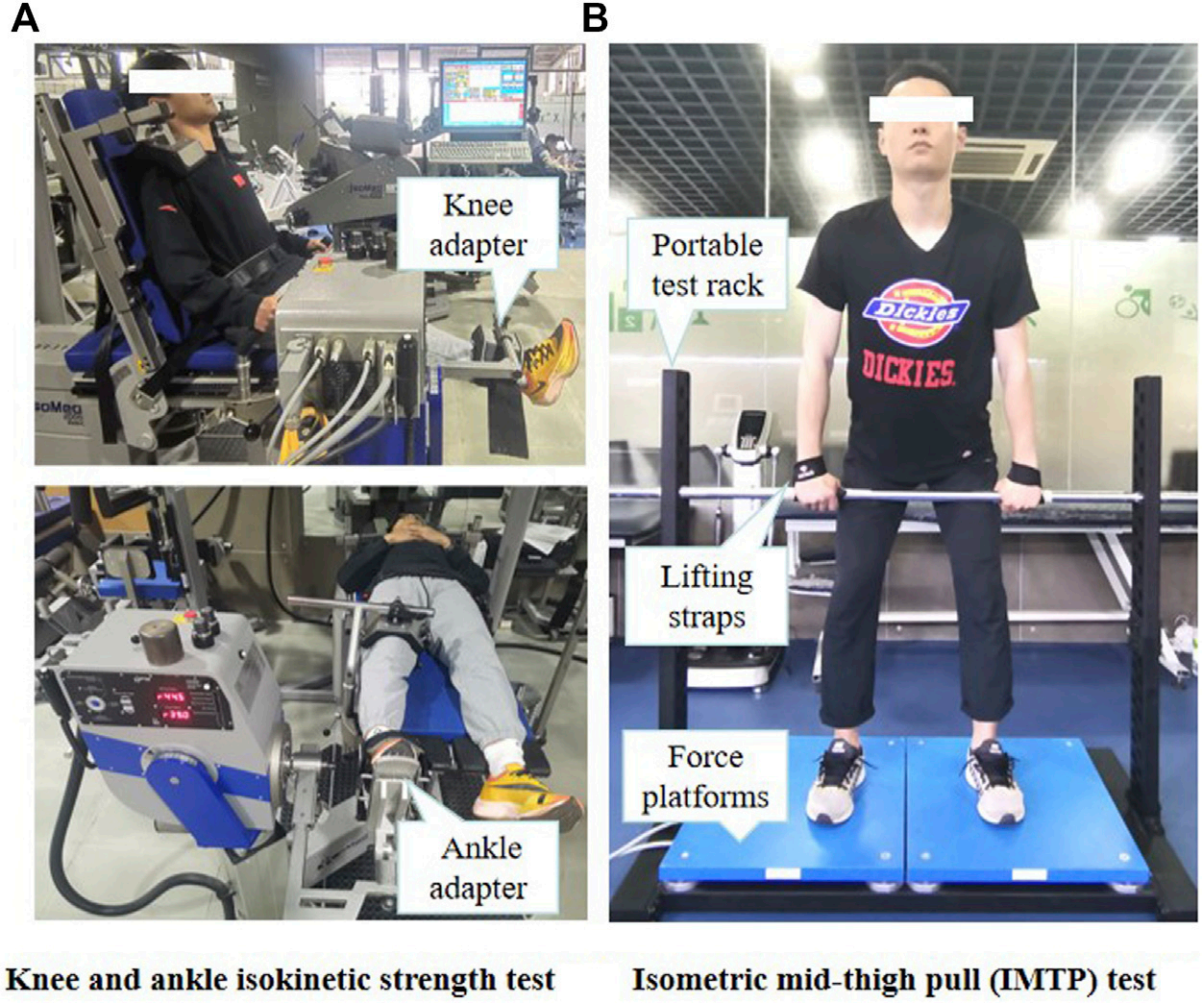
Strength test. Interpretation of **(A)** knee and ankle isokinetic strength test **(B)** Isometric mid-thigh pull test. IMTP, isometric mid-thigh pull.

Knee joint flexor/extensor muscles and ankle joint plantar flexor/dorsiflexor muscles PT were measured using a motor-driven dynamometer (D&R Ferstl GmbH, Hemau, Germany) (Figure 3A) as this test showed high ICC (knee: 0.90 to 0.96; ankle: 0.77–0.98) in previous study (Gonosova et al., 2018; Andrade et al., 2021). The subjects performed two trials to access the right lower-limb knee joint flexion/extension and ankle joint plantarflexion/dorsiflexion PT. Before each trial, the instrument was calibrated according to the manufacturer’s manual. The operator needs to place the shaft of the dynamometer in a substantially horizontal position to allow automatic gravity compensation and not to touch the dynamometer with the hands during weighing. The subject remains relaxed and free from muscle activity to eliminate the effects of gravity. All participants performed three sub-maximal trials to familiarise themselves with the test. As the most commonly used angular speed during isokinetic testing procedures is 60°s^−1^, which has been suggested to reliably and accurately evaluate maximal capacities of the muscles to produce force (Zapparoli and Riberto., 2017; Silva et al., 2018), five repetitions of concentric and eccentric isokinetic PT were evaluated on the knee and ankle joints at 60° s^−1^ in the present study (Luna et al., 2012; Andrade et al., 2021). There was a rest period of 1 min between contraction type and a 10-min break between trials. In addition, the subjects were offered encouragement to exert their maximum strength during the trial. The PT was calculated as the relative greatest torque value (maximal PT/body weight) during isokinetic concentric and eccentric phases and was collected for further analysis.

As shown in Figure 3A, In the first trial, knee strength was tested in a seated position, with hips at approximately 85° flexion. At the same time, the backrest was adjusted so that the participant could easily flex and extend the knee. The shoulders, hips, and distal femur were then immobilised with instruments and a safety belt. The right knee range of motion was programmed to start from full extension to 90° of flexion (Sundby and Gorelick, 2014). During the test, the subjects were asked to hold onto the handles to keep their posture stable. The knee flexor muscles concentric (K_flex-con_), eccentric (K_flex-ecc_), knee extensor muscles concentric (K_ex-con_), and eccentric (K_ex-ecc_) PT at 60°s^−1^were measured.

Ankle strength was measured in the second trial. The participants were tested in a supine position on the dynamometer seat with the knees and hips fully extended. The right foot was strapped to the ankle adapter of the dynamometer and fixed with a safety belt. The positions of thighs, hips, and shoulders were fixed. The shaft of the dynamometer was aligned with the axis of the lateral malleolus. The ankle neutral position was programmed to 0°, and the movement range began from 15° dorsiflexion to 40° plantar flexion (Tsiokanos et al., 2002). The ankle dorsiflexor muscles concentric (A_dors-con_), eccentric (A_dors-ecc_), plantar flexor muscles concentric (A_plan-con_), and eccentric (A_plan-ecc_) PT at 60° s^−1^were measured.

#### Isometric Mid-Thigh Pull Test

The IMTP test was performed on two force platforms sampling at 1000 HZ (9290AA; Kistler, Winterthur, Switzerland) and a portable test rack (Figure 3B). This testing has consistently been shown to be highly reliable with ICCs ranging from 0.80 to 0.97 (Mcguigan, 2019). Before the test, the subject was instructed to place and mark the crossbar in the clean second pull position, which was defined as 140–150° of hip flexion and 125–145° of knee flexion (Chavda et al., 2020). All participants were asked to use an overhand grip while secured to the barbell with lifting straps to eliminate the influence of grip strength. Four warm-up trials were performed using 50, 70, 80, and 90% of their maximum effort with a rest period of 60 s (Beattie et al., 2017). Before the trial, subjects were instructed to “Push the ground fast and hard with maximum effort” to perform to their best, followed by completing three maximal effort IMTP tests to obtain 3 force-time curves of 2 s standing still and 5 s pulling with full force. The force-time characteristics with the highest PF was included in the statistical analysis. There was a 3-min interval between trials, and the subjects were greatly encouraged in each trial.

The highest force generated during IMTP is reported as PF expressed relative to body weight. In addition, the time-specific force (∆Force) at 50–350 ms from the initiation of the pull was collected. RFD was calculated using the following equation:

RFD=ΔForce/ΔTime (4)

RFD was applied to predetermined specific time intervals: 0–50 to 0–350 ms (RFD_0–50_, RFD_0–100_, RFD_0–150_, RFD_0–200_, RFD_0–250_, RFD_0–300_, and RFD_0–350_). These time intervals were selected based on the ground-contact times during various submaximal running speeds reported earlier (Gómez-Molina et al., 2017; García-Pinillos et al., 2019a).

### Statistical Analyses

The Shapiro-Wilk test was used to test data normality. Values are expressed as mean ± SD. The correlations between the isokinetic lower-limb joint PT (knee and ankle concentric and eccentric PT), lower-limb stiffness (K_vert_, K_leg_, K_knee_, and K_ankle_), and IMTP force-time characteristics (PF and specific time RFD at 0–50 to 0–350 ms) and RE (10 km·h^−1^) were analysed using Pearson’s correlation coefficients. Correlations were classified as “small” when r = 0.1–0.3, “moderate”, when r = 0.3–0.5, “large”, when r = 0.5–0.7, “very large”, when r = 0.7–0.9, and “extremely large”, when r = 0.9–1.0 (Hopkins et al., 2009). All reported *p* values were corrected using the Benjamini–Hochberg procedure, a method used to correct for false-discovery rates arising from statistical multiple comparisons (Benjamini and Hochberg, 1995). Statistical analyses were performed using SPSS (version 22.0; IBM Corp. Armonk, NY, United States) and the statistical programming language R (www.r-project.org). Statistical significance was set at an alpha level of *p* < 0.05, for Pearson’s correlation coefficients.

## Results

All physical and physiological characteristics, lower-limb stiffness, isokinetic strength, and IMTP force-time characteristics values are shown in Table 1. The correlation coefficients between the neuromuscular indicators and RE are demonstrated in Table 2.

**TABLE 2:** Correlations between neuromuscular characteristics and RE at 10 km·h^−1^ (*n* = 30).

	RE at 10 km·h^−1^			RE at 10 km·h^−1^	
	Correction Coefficient (R)	Q Value (Corrected *p* value)		Correction Coefficient (R)	Q Value (Corrected *p* value)
**K**_**vert**_ **at 10 km**·**h** ^**−1**^	**−0.449** **^a^**	**0.049**	A_dors-ecc_ at 60°s^−1^	0.096	0.881
K_leg_ at 10 km·h^−1^	**−**0.100	0.881	A_plan-ecc_ at 60°s^−1^	**−**0.037	0.937
K_knee_ at 10 km·h^−1^	**−**0.197	0.594	PF	0.070	0.881
K_ankle_ at 10 km·h^−1^	**−**0.394	0.073	**RFD**_**0–50**_	**−0.438** **^a^**	**0.049**
K_flex-con_ at 60°s^−1^	**−**0.070	0.881	**RFD**_**0–100**_	**−0.515** **^a^**	**0.027**
K_ex-con_ at 60°s^−1^	0.132	0.881	**RFD**_**0–150**_	**−0.434** **^a^**	**0.049**
K_flex-ecc_ at 60°s^−1^	**−**0.026	0.937	**RFD**_**0–200**_	**−0.534** **^a^**	**0.027**
K_ex-ecc_ at 60°s^−1^	0.090	0.881	**RFD**_**0–250**_	**−0.527** **^a^**	**0.027**
A_dors-con_ at 60°s^−1^	**−**0.061	0.881	**RFD**_**0–300**_	**−0.441** **^a^**	**0.049**
A_plan-con_ at 60°s^−1^	0.011	0.955	RFD_0–350_	**−**0.390	0.073

a Significant correlation (*p < 0.05). Significant correlations are presented in bold letters and numbers. K_vert_, vertical stiffness; K_leg_, leg stiffness; K_knee_, knee joint stiffness; K_ankle_, ankle joint stiffness; K_flex-con_ PT, knee flexor muscles relative peak torque in concentric action; K_ex-con_ PT, knee extensor muscles relative peak torque in concentric action; K_flex-ecc_ PT, knee flexor muscles relative peak torque in eccentric action; K_ex-ecc_ PT, knee extensor muscles relative peak torque in eccentric action; A_dors-con_ PT, dorsiflexor muscles relative peak torque in concentric action; A_plan-con_ PT, plantar flexor muscles relative peak torque in concentric action; A_dors-ecc_ PT, dorsiflexor muscles relative peak torque in eccentric action; A_plan-ecc_ PT, plantar flexor muscles relative peak torque in eccentric action; PF, relative peak force; RFD, rate of force development; RFD_0–50_, RFD, 0–50 ms; RFD_0–100_, RFD, 0–100 ms; RFD_0–150_, RFD, 0–150 ms; RFD_0–200_, RFD, 0–200 ms; RFD_0–250_, RFD, 0–250 ms; RFD_0–300_, RFD, 0–300 ms; RFD_0–350_, RFD, 0–350 ms.

For isokinetic strength testing, there were non-significant, small correlations between the knee (r = −0.070 to 0.132, *p* = 0.881–0.937) and ankle joint strength (r = −0.061 to 0.096, *p* = 0.881–0.955) with RE at 10 km·h^−1^.

**FIGURE 4:**
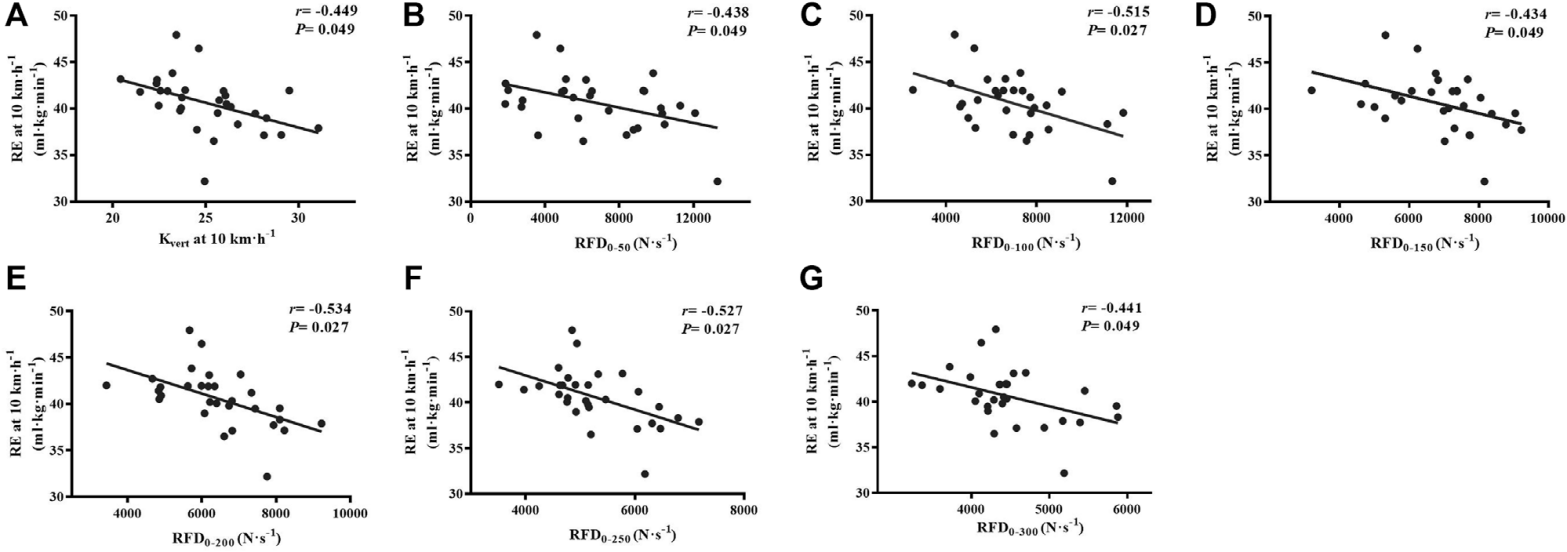
Correlations of K_vert_, IMTP force-time characteristics with 10 km·h^−1^ **(A–G)** running economy. K_vert_, vertical stiffness; RFD, rate of force development; RFD_0–50_, RFD 0–50 ms; RFD_0–100_, RFD 0–100 ms; RFD_0–150_, RFD 0–150 ms; RFD_0–200_, RFD 0–200 ms; RFD_0–250_, RFD 0–250 ms; RFD_0–300_, RFD 0–300 ms; RFD_0–350_, RFD 0–350 ms; IMTP, isometric mid-thigh pull.

With regard to lower-limb stiffness, we found that the K_vert_ (r = −0.449, *p* = 0.049) moderately correlated with RE at 10 km·h^−1^ (Figure 4). Meanwhile, non-significant, small correlations were found between K_leg_ (r = −0.100, *p* = 0.881), K_knee_ (r = −0.197, *p* = 0.594), K_ankle_ (r = −0.394, *p* = 0.073), and RE after Benjamini–Hochberg adjustment.

For the IMTP test, we found moderate to large relationships between the specific time RFD at 0–50 to 0–300 ms (r = −0.434 to −0.534, *p* < 0.05) and RE at 10 km·h^−1^ after false-discovery rate corrections (Figure 4). There were no significant relationships between the PF (r = 0.070, *p* = 0.881) and RE.

## Discussion

This study aimed to investigate the relationship between isokinetic knee and ankle joint strength, lower-limb stiffness, isometric force-time characteristics, and RE in recreationally trained male runners. Our primary findings were that knee and ankle joint strength, and stiffness were not significantly correlated with RE. The K_vert_, but not K_leg_, was significantly associated with RE. Furthermore, the time-specific RFD at 0–50 to 0–300 ms were found to be significantly correlated with RE. Non-significant, small-sized relationships were found between IMTP PF and RE at 10 km·h^−1^.

### Isokinetic Lower-Limb Joint Strength and Running Economy

The muscles around the knee and ankle joints play an important role in running. However, the results of this study reject our initial hypothesis and do not show any significant correlation between isokinetic knee and ankle muscle strength and RE. This finding is in line with that the studies of Sundby and Gorelick. (2014) and Andrade et al. (2021), who reported no correlation between concentric or eccentric knee flexion and extension PT and RE in distance runners. It is worth noting that the behavior of the knee/ankle flexor and extensor muscles during running was different from that in the isokinetic strength test. During the running support phase, muscle fascicles length changes are decoupled from tendon length changes (Farris and Sawicki, 2012). The knee extensor muscle fascicles contract quasi-isometrically while the tendon is stretched, optimising the storage and return of elastic strain energy in the tendon (Monte et al., 2020). Similarly, the ankle plantar flexor (i.e., gastrocnemius medialis) and dorsiflexor muscles fascicles (i.e., tibialis anterior) are co-activated and contracted isometrically to develop force, and absorb energy through stretching of the tendinous tissue at foot contact and returning it to the body in subsequent strides (Tam et al., 2018; Maharaj et al., 2019; Monte et al., 2020). For the isokinetic strength test, the knee flexor/extensor and ankle plantar flexor/dorsiflexor muscles are purely concentric or eccentric, and the storage of elastic strain energy is not possible because the tendon does not undergo a preceding stretch (Roberts, 2002).

Apart from the intrinsic muscle-tendon behavior, the isokinetic test performs an open chain movement, while running is a sport that combines closed and open chain movements (Andrade et al., 2021). Stensdotter et al. (2003) found differences in the EMG onset and amplitude of the different knee extensor muscles during the open and closed kinetic chain test. For example, during closed chain knee extension, the quadriceps muscles onset was almost simultaneous, whereas in the open chain the rectus femoris was activated first and the vastus medialis obliquus was activated last. Li et al. (2019) reported that isokinetic leg press tests (closed chain movement) were largely correlated with RE, indicating that the effect of movement pattern choosing in test on predicting RE. Worth to note that the angular velocity generated during running is much greater than the present isokinetic strength test (60°s^−1^). The maximum angular velocity generated by the kinematics of the joints (knee and ankle) during the human running gait cycle is between 400 and 600°s^−1^ (Grimmer and Seyfarth, 2014). Meanwhile, the absence of angular acceleration in the isokinetic test is also very distinct from the functional joint movements during running (Andrade et al., 2021). Therefore, the muscle contraction behavior, movement pattern and angular velocity differences may limit the association between isokinetic strength testing and practical running.

### Lower-Limb Stiffness and Running Economy

K_vert_ and K_leg_ as global stiffnesses describe the ability of the entire lower limb to reduce energy consumption and to utilise elastic energy during vertical and horizontal movements (Dalleau et al., 1998; Heise and Martin, 2001; Struzik et al., 2021). In our study, the K_leg_ values (13.04 kN·m^−1^) and K_vert_ values (25.07 kN·m^−1^) reported are similar to those of previous studies at similar speeds among recreational runners (Morin et al., 2005; García-Pinillos et al., 2019b). We found that K_vert_, but not K_leg_, was significantly negatively associated with RE at 10 km·h^−1^. This finding is consistent with Heise and Martrin. (1998) and partially supports our hypothesis. K_vert_ is mainly determined by the COM vertical displacement, a higher K_vert_ allowing for more economical running tasks (with less COM vertical displacement) and with superior performance through greater potential elastic energy return from the tendon structure (Struzik et al., 2021). Folland et al. (2017) reported that vertical displacement of COM (the standard measure of vertical oscillation) explained 28% of the inter-individual variation in RE at 10 and 12 km·h^−1^ across diverse competitive runners. Therefore, a higher K_vert_ may be an important contributor to regulate RE by reducing vertical oscillation during the stance phase.

Li et al. (2019) recently reported that K_leg_ has a large correlation with RE at 12–16 km·h^−1^, which indicates that K_leg_ is an essential determinant of RE in well-trained runners. However, this is not consistent with our findings. The difference in K_leg_ may be due to the experience of endurance running training. For runners, their energy consumption has a curvilinear U-shaped relationship with K_leg_ rather than a linear one, and there is an optimal K_leg_ to minimise energy costs (Hunter and Smith, 2007; Moore et al., 2019). Highly trained runners are able to quickly adapt to the optimal regulation of K_leg_ through prolonged running experience, such as changing gait characteristics (e.g., stride length, stride frequency and contact time) to improving RE (Hunter and Smith, 2007; Moore et al., 2019). In contrast, no optimisation of the above K_leg_ or gait characteristics was found in recreational runners, who were further away from their optimal stiffness than trained runners (De Ruiter et al., 2014; Bitchell et al., 2019). Moreover, the percentage contribution of elastic strain energy to positive work was shown to be reduced at lower running speeds (Lai et al., 2014). Therefore, it may not be the most advantageous condition for our recreational subjects to improve RE through the reuse of elastic energy in the lower limbs at slow running speeds (i.e., 10 km·h^−1^).

Joint stiffness reflects the elastic properties of the musculotendinous tissue surrounding individual joint (Kuitunen et al., 2002). Tam et al. (2018) reported that knee and ankle stiffness were significantly associated with RE in trained runners. However, contrary to our initial hypothesis, our results do not exhibit this significant relationship in recreational runners. Many studies indicate that changes in joint stiffness partly be related to foot strike pattern during landing phase (Butler et al., 2003). Compared to a rearfoot strike pattern, knee stiffness was greater and ankle stiffness was lower in the forefoot strike pattern (Hamill et al., 2014). In our study, all subjects were rearfoot strike pattern, whereas Tam et al. (2018) did not report the foot strike pattern of their subjects landed. Considering that the highly trained runners may possibly use the midfoot or forefoot landing pattern (Hasegawa et al., 2007; Mo et al., 2021), thus resulting in the different values of joint stiffness. In addition, the running speed affects joint stiffness. Kuitunen et al. (2002) reported increasing running velocity was associated with increases in knee stiffness. Specifically, Tam et al. (2018) determined K_Knee_ and K_ankle_ using the running speed of 3.28 m·s^−1^ while our study using of 2.78 m·s^−1^. Future studies should ascertain if ankle and knee stiffness would be influence at different running speeds.

Although we did not observe K_leg_, K_knee_ and K_ankle_ association with RE at 10 km·h^−1^, previous studies indicate the significant contributions to running performance especially in well-trained runners, as elastic energy accumulation is an important factor in reducing energy costs (Struzik et al., 2021). Therefore, strategies to improve RE through the reuse of elastic energy may be more advantageous for recreational runners at higher running speeds. At same time, lower-limb muscles also need to shorten at faster velocities and recruit more motor units to generate the necessary high forces in a very short contact time (Fletcher and MacIntosh, 2017).

### Isometric Force-Time Characteristics and Running Economy

IMTP is performed to measure the force-time characteristics (i.e., PF and RFD) of various sports (Mcguigan, 2019), and allows the hip, knee, and ankle joints to be at a relatively biomechanical angle during running (Lum et al., 2020). Lum et al. (2020) recently reported that the IMTP time-specific RFD (0–100 to 0–200 ms) significantly correlate with RE at submaximal speeds, indicating that runners with higher force production exhibit better RE. However, the authors did not evaluate forces above 200 ms in their study. Our data (252 ms), as well as previous studies (Gómez-Molina et al., 2017; García-Pinillos et al., 2019a), suggest that ground contact time is greater than 200 ms at running speeds of 10 km·h^−1^. Therefore, it is essential to evaluate forces above 200 ms for recreational runners. As hypothesised, RFD within the running contact time was significantly correlated with RE. In the present study, we found that time-specific RFD (0–50 to 0–300 ms) with 10 km·h^−1^ RE were moderate to largely inversely correlated. The reasons for this phenomenon can be explained as follows. Firstly, from the behavior of the lower limb muscle during the support period, a larger RFD may allow the lower-limb muscle to activate rapidly or generate higher forces during a shorter contact time (Lum et al., 2020), promoting favourable muscles conditions (quasi-isometric contraction and near-optimal length), thus optimising the storage and return of elastic energy and reducing the extra work done by the muscles (Lai et al., 2014; Fletcher and MacIntosh, 2017; Bohm et al., 2019; Monte et al., 2020). Secondly, the greater RFD theoretically enables the runners to rapidly push off the ground, thereby decreasing ground contact and muscle contraction time during the stance phase, which results in a more rapid transition of the running gait from the braking to the propulsive phase (Lum et al., 2020). This may potentially reduce vertical oscillation and metabolic demands against gravity (Saunders et al., 2004; Lima and Blagrove, 2020).

Muscles contracting to produce high force theoretically allow the runners to maintain a given speed or perform every running action at a relatively lower intensity (Fletcher and MacIntosh, 2017). However, contrary to our initial hypothesis, we did not find a significant relationship between IMTP PF and RE. This finding is consistent with those of Lum et al. (2020), who reported that PF is not significantly related to RE or running performance in male runners. This can be attributed to the fact that the muscular behavioral processes that drive energy savings need to be timed precisely during the support phase of running (Barnes and Kilding, 2015), whereas the IMTP test require a relatively long time for runners to reach peak force (Chavda et al., 2020). Therefore, compared to the time-specific RFD, the PF is not specific in predicting the energetic cost of running.

This study has certain limitations that must be acknowledged. Firstly, the current findings indicate that the neuromuscular characteristics mentioned above is an acceptable predictor of RE in recreational male runners. Further studies are needed to investigate the potential relationships among elite runners and female runners’ cohort. Secondly, we only test isokinetic joint strength at 60°s^−1^ velocity, as kinematics of the joints (knee and ankle) produces maximum angular velocities between 400 and 600°s^−1^ during the human running gait cycle, it is valuable to investigate the relationship between joint strength and RE at higher angular velocities. Finally, a large correlation does not imply a cause-effect relationship. Hence, in the future, studies with longitudinal study design should be performed to confirm the effects of isometric force-time characteristics, lower-limb stiffness and their manipulation through intervention on running economy.

## Conclusion

In summary, we did not find significant correlations between knee and ankle joint strength, stiffness, and RE. However, for the whole lower-limb neuromuscular characteristics, IMTP time-specific RFD at 0–50 to 0–300 ms and vertical stiffness significantly correlated with RE at 10 km·h^−1^ in recreational runners.

Although isokinetic testing is the gold standard for measuring muscle strength (Kambič et al., 2020), given the specific movement of running, the joint strength at higher angular velocity (i.e., 240°s^−1^) and multi-joint closed-chain movement (i.e., leg press) need be considered when measuring the strength capacity of runners. In addition, since the lower limb muscles preform isometric contractions during the running contact phase, the isometric contraction mode should be utilized in the isokinetic strength test. When evaluating lower limb stiffness, the global stiffness (i.e., vertical or leg stiffness) takes precedence over local stiffness (i.e., ankle or knee joint stiffness) to evaluate runners’ elastic energy utilization. Finally, the results also suggest that runner should focus on ability to rapidly generate force which correspond to the foot contact time (<300 ms).

## Data Availability

The raw data supporting the conclusions of this article will be made available by the authors, without undue reservation.
